# Values of a novel comprehensive prognostic nutritional index (FIDA) in the prognosis of non-small cell lung cancer

**DOI:** 10.3389/fonc.2024.1393684

**Published:** 2024-06-20

**Authors:** Han Qiao, Yan Feng, Xiaolei Han, Huaping Tang

**Affiliations:** ^1^ Department of Respiratory Medicine, Qingdao University, Qingdao, China; ^2^ Department of Health Office, Qingdao Municipal Hospital, Qingdao, China; ^3^ Department of Respiratory Medicine, Qingdao Municipal Hospital, Qingdao, China

**Keywords:** non-small-cell lung carcinoma (NSCLC), FIDA, nomogram, prognosis, overall survival (OS)

## Abstract

**Background:**

This study focuses on determining the prognostic and predictive value of the comprehensive prognostic nutrition index (FIDA) in individuals undergoing treatment for Non-Small-Cell Lung Carcinoma (NSCLC).

**Methods:**

This retrospective analysis encompassed 474 of NSCLC patients treated from January 2010 through December 2019. Employing the Lasso-COX regression approach, eight blood parameters were identified as significant prognostic indicators. These parameters contributed to the formulation of the comprehensive prognostic nutrition index FIDA. Utilizing X-tile software, the patient cohort was categorized into either a high or low FIDA group based on an established optimal threshold. The cohort was then randomly segmented into a training set and a validation set using SPSS software. Subsequent steps involved conducting univariate and multivariate regression analyze to develop a prognostic nomogram. The effectiveness of this nomogram was evaluated by calculating the AUC.

**Results:**

Analysis of survival curves for both the training and validation sets revealed a poorer prognosis in the high FIDA group compared to the low FIDA group. This trend persisted across various subgroups, including gender, age, and smoking history, with a statistical significance (p<0.05). Time-dependent ROC and diagnostic ROC analyses affirmed that FIDA serves as an effective diagnostic and prognostic marker in NSCLC. Moreover, Cox regression multivariate analysis established FIDA as an independent prognostic factor for NSCLC. The prognostic nomogram, integrating FIDA and clinical data, demonstrated substantial prognostic utility and outperformed the traditional TNM staging systemin predicting overall survival (OS).

**Conclusion:**

FIDA emerges as a dependable predictor of outcomes for patients with NSCLC. It offers a practical, cost-effective tool for prognostication in regular clinical applications.

## Introduction

Cancer continues to be the primary cause of mortality globally (1, 2). Among various types, lung cancer, particularly NSCLC, ranks as the most prevalent (3). While patients in early stages have the option of standard surgical interventions, the subtle clinical symptoms and signs often result in the diagnosis of advanced-stage tumors at initial detection, leading to a generally poor prognosis for these patients (4). Consequently, the discovery and validation of effective prognostic biomarkers are crucial in refining treatment approaches and enhancing the long-term survival prospects of these patients (5).

The inflammatory response is crucial in various phases of tumor progression (6), with neutrophils significantly influencing tumor-related inflammation. They contribute to this process by generating chemokines and cytokines that suppress lymphocyte immunoreactivity, a factor intimately linked to tumor cell metastasis (7). Moreover, abnormalities in coagulation markers are observed in approximately 50% of cancer patients, rising to 90% in those with metastasis (8). Notably, this includes elevated fibrinogen (Fib) and fibrin degradation products, along with variable increases in D-dimer (D-D) levels (9). D-D levels, in particular, are known to vary significantly in the early tumor stages and have been acknowledged as an additional prognostic indicator in cancers like breast, ovarian, and colon (10). Recent studies have highlighted the prognostic significance of new inflammatory markers that integrate traditional parameters. These include the ratio of neutrophil count to lymphocyte count (NLR) (11), ratio of platelet count to lymphocyte count (PLR) (12), ratio of lymphocyte count to monocyte count (LMR) (13), ratio of monocyte count to lymphocyte count (MLR) (14), serum albumin + 5*total lymphocyte (OPNI) (15), platelet count * neutrophil count ratio to lymphocyte count (SII) (16), monocyte count * neutrophil count to lymphocyte count (SIRI) (17), reflecting both nutritional and inflammatory states. However, the efficacy of combining inflammatory and nutritional variables in predicting lung cancer prognosis remains uncertain. Therefore, our retrospective cohort study aimed to identify specific prognostic factors in lung cancer by examining demographic data, etiology, and clinical features. We developed and validated a predictive model by integrating these variables, offering a tool for prognostic prediction and treatment options in clinical practice.

## Materials and methods

### Subject investigated

This retrospective study encompassed patient data from Qingdao Municipal Hospital, specifically targeting those diagnosed with NSCLC between January 2010 and December 2019. The 8th AJCC/UICC TNM staging system was carried out for the current study. Criteria for inclusion comprised (1): NSCLC confirmed via cytology or pathology in our institution (2); availability of comprehensive clinical, pathological, and imaging records (3); received first-line treatment, including surgery, chemotherapy, or targeted therapy. The study excluded participants if they had (1): recent infections or anti-infective treatments within a month prior to enrollment (2); severe cardiac, hepatic, renal, or hematologic conditions (3); concurrent malignancies. Ultimately, the research included 474 patients. Adhering to the Declaration of Helsinki’s guidelines, the study protocol received approval from the Ethics Committee of Qingdao Municipal Hospital(2024-LW-008). All retrospective data including in this study was anonymous, so the requirement of informed consent for this retrospective study was waived.

### Data collection

Clinical data and laboratory data were collected using routine blood tests for fibrinogen (Fib), white blood cells (WBC), neutrophils (N), lymphocytes (L), monocytes (M), D dimer (D-D), platelets (PLT), hemoglobin (Hb), albumin (ALB), globulin (GLB), prealbumin (PAB) within 1 week before diagnosis. The ratio of albumin to globulin (AGR) (18), NLR, derived neutrophil to lymphocyte ratio (dNLR) (19), PLR, LMR, MLR, OPNI, SII, and SIRI were calculated.

### Case follow-up

The observation period for this study commenced on the date each patient was diagnosed and concluded on December 31, 2021. Monitoring methods encompassed both in-person visits, either as outpatient or during hospital admissions, and telephonic check-ins. These follow-up assessments focused on monitoring subsequent treatments, evaluating treatment outcomes, tracking relapse occurrences, and recording the time of death. The definition of overall survival in this context was the duration from the date of diagnosis to either the date of death from any cause or the date of the last follow-up. The median duration of follow-up was established at 42.57 months. By the end of this period, 201 patients, accounting for 42.4% of the cohort, had passed away.

### Statistical analysis

The study employed a LASSO Cox regression analysis through the “glmnet” package in R software to extract the most impactful hematological prognostic indicators from variables. For determining the optimal threshold of FIDA, the X-tile software was utilized. Patient randomization into training and validation cohorts was conducted using SPSS. The influence of varying FIDA groups on OS was evaluated with Kaplan-Meier survival plots and log-rank testing. The “timeROC” package in R was used for creating transient ROC curves and computing time-dependent ROC metrics, with 1-year, 3-year, and 5-year AUC values being derived for these curves. HR along with 95% confidence intervals (95% CI) were deduced through Cox proportional hazard regression modeling. The identification of independent prognostic factors involved both univariate and multivariate COX regression analyses. The study also incorporated discriminant and calibration assessments from both training and validation datasets. Calibration curves were employed for visual representation, and the discriminative efficacy of nomogram was contrasted against the traditional TNM staging systemin by calculating their AUC. Data processing and graphical representations were executed using SPSS version 26.0.1, Graph Pad Prism 8.1.0, and R version 4.2.1. Statistical significance was assigned to differences where P < 0.05.

## Results

### Baseline characteristics of patients

In this investigation, 474 lung cancer patients were deemed eligible and subsequently included (**Table 1**). They were divided into two groups: a training cohort (comprising 330 patients) and a validation cohort (consisting of 144 patients), following 7:3 distribution. The training cohort predominantly comprised males (206 patients, representing 62.4%) and females (124 patients, accounting for 37.6%), with their median age being 62 years (interquartile range: 55 to 67 years). The most common cancer type in this cohort was adenocarcinoma, encompassing 80.6% of cases, and a notable proportion of these patients had a history of smoking (45.5%). In the validation cohort, males constituted 53.5% (77 patients), with a median age closely matching that of the training cohort, at 62 years (interquartile range: 55 to 66 years). Based on the criteria of the 8th edition AJCC TNM staging system, there were 119 (36.1%), 54 (16.4%), 71(21.5%) and 86 (26.1%) cases in stage I, II, III and IV in the training cohort and 57 (39.6%), 17 (11.8%), 34(23.6%) and 36 (25%) cases in the validation cohort, respectively. When comparing both clinical indicators across both cohorts, no significant statistical differences were observed (p>0.05).

**Table 1:** Baseline characteristics of patients.

Characteristics	Validation Set	Training Set	Total Set	P value
n	144	330	474	
Sex, n (%)				P=0.188
Female	67 (46.5%)	124 (37.6%)	191 (40.3%)	
Male	77 (53.5%)	206 (62.4%)	283 (59.7%)	
Age(years), median (IQR)	62 (56, 66)	62 (55, 67)	62 (56, 66)	P=0.986
History of smoking, n (%)				P=0.341
No	89 (61.8%)	180 (54.5%)	269 (56.8%)	
Yes	55 (38.2%)	150 (45.5%)	205 (43.2%)	
Pathological classification, n (%)				P=0.937
Adenocarcinoma	114 (79.2%)	266 (80.6%)	380 (80.2%)	
Non-adenocarcinoma	30 (20.8%)	64 (19.4%)	94 (18.8%)	
Tumor location, n (%)				P=0.416
U	73 (50.7%)	189 (57.3%)	262 (55.3%)	
M/L	71 (49.3%)	141 (42.7%)	212 (44.7%)	
Tumor diameter(cm), median (IQR)	3 (1.5, 4.675)	3 (1.8, 5)	3 (1.5, 4.975)	P=0.661
AJCC TNM Stage, n (%)				P=0.923
I	57 (39.6%)	119 (36.1%)	176 (37.1%)	
II	17 (11.8%)	54 (16.4%)	71 (15%)	
III	34 (23.6%)	71 (21.5%)	105 (22.2%)	
IV	36 (25%)	86 (26.1%)	122 (25.7%)	
Metastasis, n (%)				P=0.953
No	108 (75%)	243 (73.6%)	351 (74.1%)	
Yes	36 (25%)	87 (26.4%)	123 (25.9%)	
ECOG-PS, n (%)				P=0.930
0–1	108 (75%)	242 (73.3%)	350 (73.8%)	
≥2	36 (25%)	88 (26.7%)	124 (26.2%)	
D-D(0–0.5), ug/ml	0.485 (0.3075, 0.865)	0.44 (0.29, 0.7375)	0.45 (0.2925, 0.7675)	P=0.412
Fib(2–4), g/l	3.28 (2.82, 4.235)	3.31 (2.73, 4.3175)	3.305 (2.7625, 4.31)	P=0.96
WBC(3.5–9.5), 10^9/l	6.395 (5.2075, 8.165)	6.51 (5.3725, 8.2875)	6.49 (5.3, 8.225)	P=0.776
N(1.8–6.3), 10^9/l	3.85 (2.7075, 5.235)	3.94 (2.98, 5.12)	3.915 (2.9, 5.1275)	P=0.73
L(1.1–3.2), 10^9/l	1.895 (1.5675, 2.3025)	1.865 (1.4425, 2.32)	1.88 (1.4725, 2.3175)	P=0.811
M(0.1–0.6), 10^9/l	0.425 (0.31, 0.5425)	0.43 (0.33, 0.56)	0.43 (0.32, 0.5575)	P=0.987
PLT(125–135), 10^9/l	242 (205, 288.75)	232.5 (187.25, 293.75)	236 (191.5, 292)	P=0.6
Hb(130–175), g/l	133 (124, 142.25)	137 (124, 146)	136 (124, 145)	P=0.396
ALB(40–55), g/l	39.065 (36.27, 42.425)	39.815 (36.555, 42.71)	39.66 (36.49, 42.662)	P=0.888
GLB(20–40), g/l	29.41 (26.447, 32.748)	28.63 (25.672, 32.558)	28.935 (25.973, 32.697)	P=0.411
PAB(200–430), mg/l	242.56 ± 62.004	251.68 ± 72.548	248.91 ± 69.576	P=0.423
LMR, median (IQR)	4.4486 (3.3984, 6.2274)	4.4083 (3.0045, 6.2514)	4.4328 (3.0627, 6.2536)	P=0.889
AGR, median (IQR)	1.3534 (1.1523, 1.5239)	1.3821 (1.2155, 1.5736)	1.3724 (1.189, 1.5533)	P=0.399
SII, median (IQR)	437.57 (323.79, 733.97)	478.21 (319.28, 811.2)	465.31 (320.28, 789.51)	P=0.951
SIRI, median (IQR)	0.83038 (0.49043, 1.4242)	0.86185 (0.49474, 1.6553)	0.85188 (0.49319, 1.5603)	P=0.791
OPNI, median (IQR)	49.05 (45.133, 53.1)	49.32 (44.425, 53.722)	49.26 (44.665, 53.333)	P=0.999
MLR, median (IQR)	0.22467 (0.16058, 0.29132)	0.22598 (0.15997, 0.33022)	0.22479 (0.15991, 0.32117)	P=0.84
NLR, median (IQR)	2.0159 (1.3923, 2.9461)	2.0349 (1.4725, 3.0559)	2.0285 (1.4377, 2.9981)	P=0.666
PLR, median (IQR)	128.67 (102.7, 162.94)	126.83 (93.9, 170.23)	127.16 (95.381, 167.85)	P=0.891
dNLR, median (IQR)	1.4295 (1.0717, 2.1031)	1.529 (1.1418, 2.1625)	1.5045 (1.1171, 2.1414)	P=0.333

### Construct the joint index FIDA

**Figure 1:**
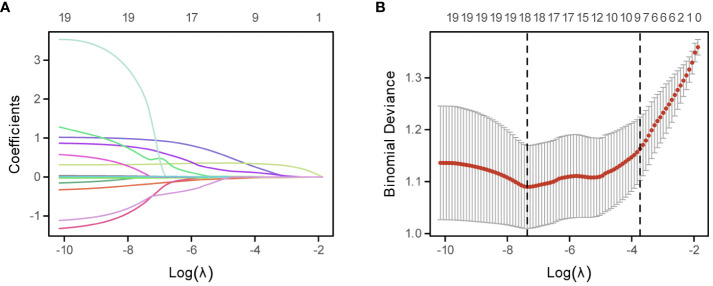
Development of the FIDA Index Using the LASSO Cox Regression Model. **(A)** Depicts the LASSO coefficient profiles of the 20 evaluated inflammatory nutritional biomarkers. Each line represents the evolution of a coefficient for an individual biomarker across the model’s iterations. **(B)** Illustrates the optimization of the LASSO model parameters, the dotted line on the right in **(B)** shows the model’s lambda value of the optimal value of the standard error of the evaluation metric 1.

To assess the effect of blood-related markers on the prognosis of lung cancer, we constructed a LASSO Cox regression model to screen the variables to reduce the overfitting of the multifactorial model. 8 variables (Fib, D-D, N, ALB, M, PLR, PAB, and WBC) were screened from 20 variables with non-zero coefficients (**Figure 1A**), and conducted through a 10-fold cross-validation process, ensuring the robustness and reliability of the model in selecting the most significant prognostic biomarkers (**Figure 1B**). The formula for calculating FIDA is as follows: FIDA = (0.333778306 × Fib) + (0.185911651 × D-D) + (0.095312555 × N) – (0.025685873 × ALB) + (0.002436591 × M) + (0.001160375 × PLR) – (0.0005955 × PAB) + (0.000167689 × WBC). Utilizing the optimal threshold derived from X-tile software (FIDA = 1.0), patients were categorized into two groups: those with a low FIDA (FIDA < 1.0) and those with a high FIDA (FIDA ≥ 1.0), as shown in **Table 2**. Within the training group, 224 patients (67.9%) fell into the low FIDA category, whereas 106 patients (32.1%) were classified in the high FIDA category. The validation group comprised 56 patients (38.9%) with high FIDA and 88 patients (61.1%) with low FIDA. Notable differences between the training and validation cohorts were observed in variables such as sex (P=0.017, P=0.006), smoking history (P=0.01, P=0.007), pathological classification (P<0.001, P<0.001), tumor diameter (P<0.001, P<0.001), AJCC TNM stage (P<0.001, P<0.001), metastasis (P<0.001, P<0.001) and ECOG-PS (P<0.001, P=0.006).

**Table 2:** Information on patients grouped by high and low FIDA in the training and validation sets.

Characteristics	Training Set			Validation Set		
	FIDA (<1.0)	FIDA (≥1.0)	p value	FIDA (<1.0)	FIDA (≥1.0)	p value
n	224	106		88	56	
Sex			**P=0.017**			**P=0.006**
Female	94 (42%)	30 (28.3%)		49 (55.7%)	18 (32.1%)	
Male	130 (58%)	76 (71.7%)		39 (44.3%)	38 (67.9%)	
Age(years)			**P=0.003**			P=0.846
<60	106 (47.3%)	32 (30.2%)		36 (40.9%)	22 (39.3%)	
≥60	118 (52.7%)	74 (69.8%)		52 (59.1%)	34 (60.7%)	
History of smoking			**P=0.01**			**P=0.007**
No	133 (59.4%)	47 (44.3%)		62 (70.5%)	27 (48.2%)	
Yes	91 (40.6%)	59 (55.7%)		26 (29.5%)	29 (51.8%)	
Pathological classification			**P< 0.001**			**P< 0.001**
Adenocarcinoma	195 (87.1%)	71 (67%)		78 (88.6%)	36 (64.3%)	
Non-adenocarcinoma	29 (12.9%)	35 (33%)		10 (11.4%)	20 (35.7%)	
Tumor location			P=0.307			P=0.247
U	124 (55.4%)	65 (61.3%)		48 (54.5%)	25 (44.6%)	
M/L	100 (44.6%)	41 (38.7%)		40 (45.5%)	31 (55.4%)	
Tumor diameter (cm)			**P<0.001**			**P<0.001**
<3.0	140 (62.5%)	19 (17.9%)		53 (60.2%)	15 (26.8%)	
≥3.0	84 (37.5%)	87 (82.1%)		35 (39.8%)	41 (73.2%)	
AJCC TNM Stage			**P<0.001**			**P<0.001**
I	105 (46.9%)	14 (13.2%)		48 (54.5%)	9 (16.1%)	
II	40 (17.9%)	14 (13.2%)		15 (17%)	2 (3.6%)	
III	46 (20.5%)	25 (23.6%)		14 (15.9%)	20 (35.7%)	
IV	33 (14.7%)	53 (50%)		11 (12.5%)	25 (44.6%)	
Metastasis			**P<0.001**			**P<0.001**
No	190 (84.8%)	53 (50%)		77 (87.5%)	31 (55.4%)	
Yes	34 (15.2%)	53 (50%)		11 (12.5%)	25 (44.6%)	
ECOG-PS			**P<0.001**			**P=0.006**
0–1	178 (53.9%)	64 (19.4%)		73 (50.7%)	35 (24.3%)	
≥2	46 (13.9%)	42 (12.7%)		15 (10.4%)	21 (14.6%)	

P< 0.05 are in bold.

### The relationship between FIDA and patient outcomes

**Figure 2:**
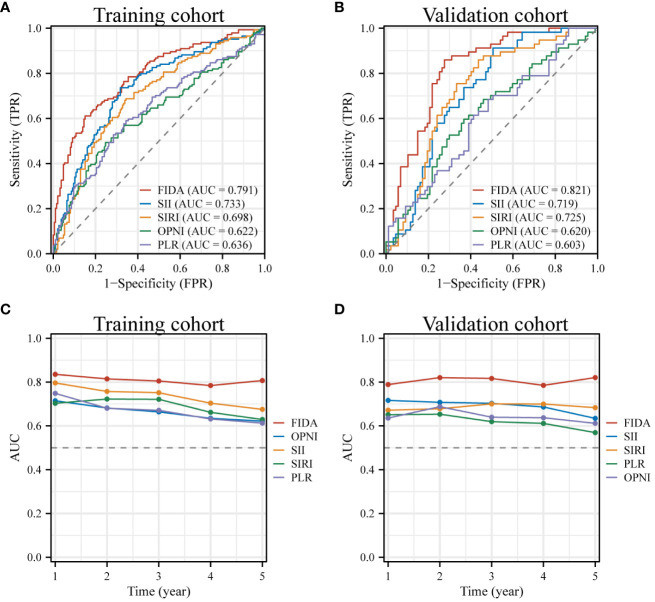
ROC Curves for Diagnosis and Prognosis in Different FIDA Groups. **(A, B)** These panels display the diagnostic ROC curves for all patients within both the training and validation sets. **(C, D)** Show the ROC curves illustrating the prognostic predictions for patients in the training and validation sets, providing insights into the effectiveness of FIDA in differentiating patient outcomes.

The analysis of the ROC curve revealed that the AUC value of FIDA in the training cohort was 0.791, which was significantly higher than SII (P=0.017, AUC=0.733), SIRI (P<0.001, AUC= 0.698), OPNI (P<0.001, AUC= 0.622), and PLR (P<0.001, AUC = 0.636) (**Figure 2A**). Similarly, the AUC value of FIDA in the validation cohort was 0.821, which was significantly higher than that of SII (P=0.0012, AUC=0.719), SIRI (P=0.0048, AUC= 0.725), OPNI (P<0.001, AUC= 0.620) and PLR (P<0.001, P<0.001). AUC = 0.603) (**Figure 2B**). Subsequently, time-dependent ROC curves were constructed to predict the prognosis of 1-, 3- and 5-year. The AUC values of the training cohort were 0.836, 0.805, and 0.807, respectively, and the AUC values of the validation cohort were 0.789, 0.817, and 0.821, respectively, all higher than other indicators (**Figures 2C, D**).These results indicated that the predictive power of FIDA for prognosis was superior to other indicators.

**Figure 3:**
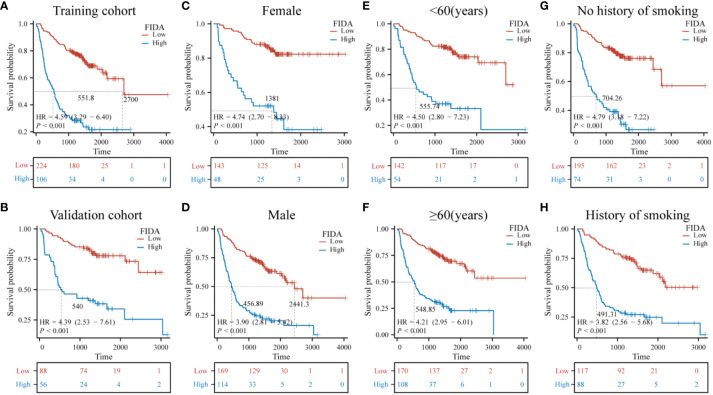
Kaplan-Meier survival curves for predicting OS in NSCLC patients in the subgroups. Kaplan-Meier survival analysis was used to compare the overall survival (OS) of the high and low FIDA groups in **(A, B)** training and validation subgroups, **(C, D)** sex (female, male), **(E, F)** age (<60, ≥60) subgroups, and **(G, H)** smoking (yes, no). FIDA was categorized into high and low groups according to the optimal threshold: red curve for low FIDA group and blue curve for high FIDA group. The dashed line indicates the median OS time. Unit: days.

**Figure 4:**
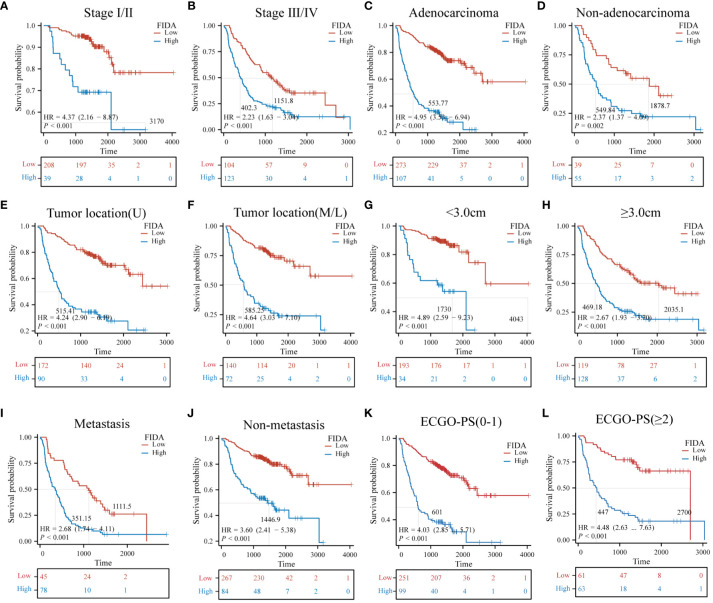
Kaplan-Meier survival curves for predicting OS in NSCLC patients in subgroups. Kaplan-Meier survival analysis was used to compare **(A, B)** stage (I/II, III/IV) subgroups, **(C, D)** pathological classification (adenocarcinoma, non-adenocarcinoma) subgroups, **(E, F)** tumor site of origin (upper lobe, middle and lower lobe) subgroups, **(G, H)** tumor size (<3.0 cm, ≥3.0 cm) subgroups, **(I, J)** metastasis (yes, no) subgroups, **(K, L)** ECGO-PS (0–1, ≥2) subgroups, Unit: days.

Furthermore, the Kaplan-Meier survival curves indicated significantly lower OS in patients with high FIDA in both cohorts (**Figures 3A, B**, P < 0.001).To more effectively evaluate the prognostic capability of the FIDA risk score, a detailed stratified analysis was carried out. This analysis was designed to verify the score’s ability to predict OS across different patient subgroups. It was observed that, in both female and male patients, those with higher FIDA levels experienced worse OS compared to the lower FIDA group (**Figures 3C, D**, P < 0.001). This pattern was consistent across various age groups, including younger (<60 years) and older (≥60 years) patients (**Figures 3E, F**, P < 0.001), as well as patients with or without a history of smoking (**Figuress 3G, H**, P < 0.001), stage I/II and III/IV groups (**Figures 4A, B**, P < 0.001), different pathological subgroups (**Figures 4C, D**, P < 0.01), different tumor site grouping (**Figures 4E, F**, P <0.001), tumor size grouping (**Figures 4G, H**, P < 0.001), whether metastasis grouping (**Figures 4I, J**, P < 0.001), and whether different ECOG-PS grouping (**Figures 4K, L**, P < 0.001).

Next, the characteristics and tumor related factors included in the patients with univariate analysis. The results showed that the factors such as Sex, Age, History of smoking, Pathological classification, Tumor diameter, AJCC TNM Stage, Metastasis, ECOG-PS, and FIDA levels were all linked to OS (**Table 3**). Subsequently, the above factors were included in a multivariate Cox regression analysis. The multivariate analysis identified several independent prognostic factors for lung cancer patients: Sex (P=0.009), AJCC TNM Stage (P<0.001), Metastasis (P <0.001), and FIDA level (P <0.001) (**Table 3**).

**Table 3:** Univariate and multivariate COX analysis of OS in training cohort.

Characteristics	Total(N)	Univariate analysis		Multivariate analysis	
		HR (95% CI)	P value	HR (95% CI)	P value
Sex	330				
Female	124	Reference		Reference	
Male	206	1.931 (1.332 - 2.799)	**P< 0.001**	1.926 (1.174 - 3.160)	**P=0.009**
Age(years)	330				
≥60	192	Reference		Reference	
<60	138	0.617 (0.436 - 0.873)	**P=0.006**	0.787 (0.549 - 1.128)	P=0.192
History of smoking	330				
No	180	Reference		Reference	
Yes	150	1.413 (1.019 - 1.961)	**P=0.038**	0.842 (0.543 - 1.304)	P=0.441
Pathological classification	330				
Adenocarcinoma	266	Reference		Reference	
Non-adenocarcinoma	64	2.055 (1.433 - 2.947)	**P< 0.001**	0.835 (0.546 - 1.276)	P=0.405
Tumor location	330				
U	189	Reference			
M/L	141	0.861 (0.617 - 1.203)	P=0.381		
Tumor diameter (cm)	330				
≥3.0	171	Reference		Reference	
<3.0	159	0.185 (0.123 - 0.281)	**P< 0.001**	0.681 (0.424 - 1.095)	P=0.113
AJCC TNM Stage	330				
I/II	119	Reference		Reference	
III/IV	211	14.463 (7.080 - 29.546)	**P< 0.001**	6.927 (3.152 - 15.223)	**P< 0.001**
Metastasis	330				
No	243	Reference		Reference	
Yes	87	5.566 (3.987 - 7.770)	**P< 0.001**	2.213 (1.535 - 3.192)	**P< 0.001**
ECGO-PS	330				
0–1	242	Reference		Reference	
≥2	88	1.708 (1.212 - 2.409)	**P=0.002**	0.999 (0.697 - 1.431)	P=0.995
FIDA	330				
<1.0	224	Reference		Reference	
≥1.0	106	4.591 (3.291 - 6.403)	**P< 0.001**	2.443 (1.661 - 3.592)	**P< 0.001**

P< 0.05 are in bold.

### Build and evaluate the prognostic nomogram

**Figure 5:**
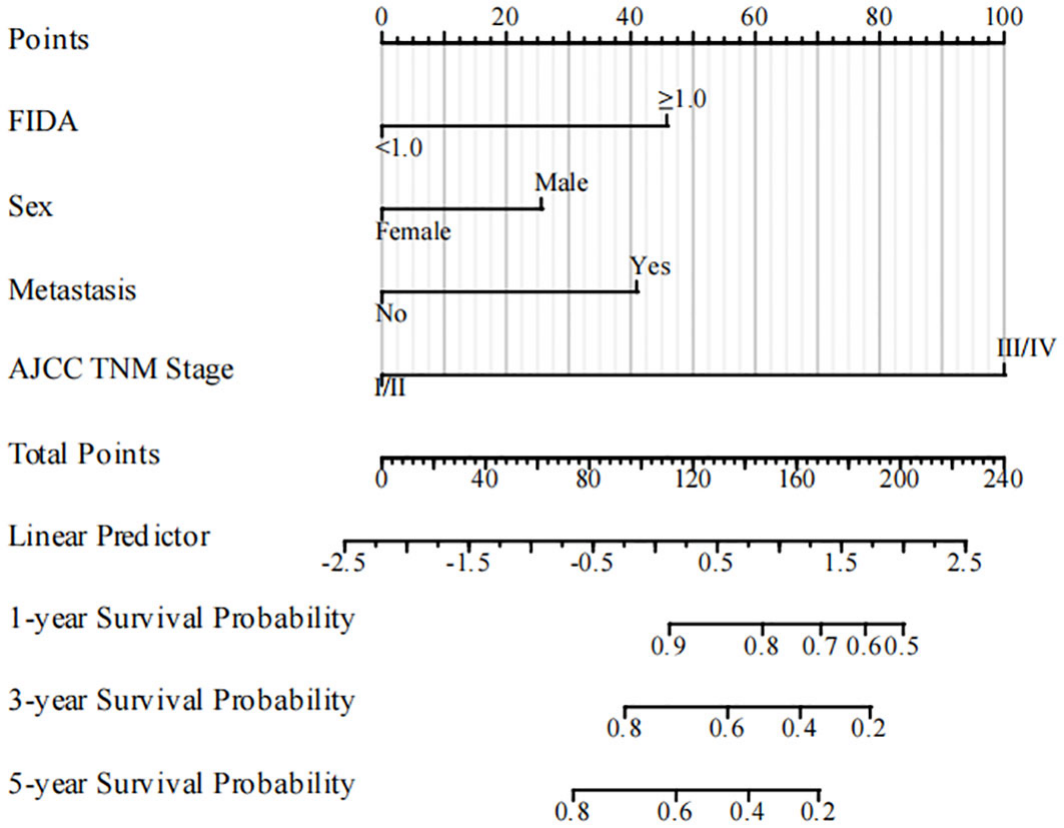
The nomogram for predicting OS rates in NSCLC patients in the training cohort. NSCLC, non-small cell lung cancer; OS, overall survival.

**Figure 6:**
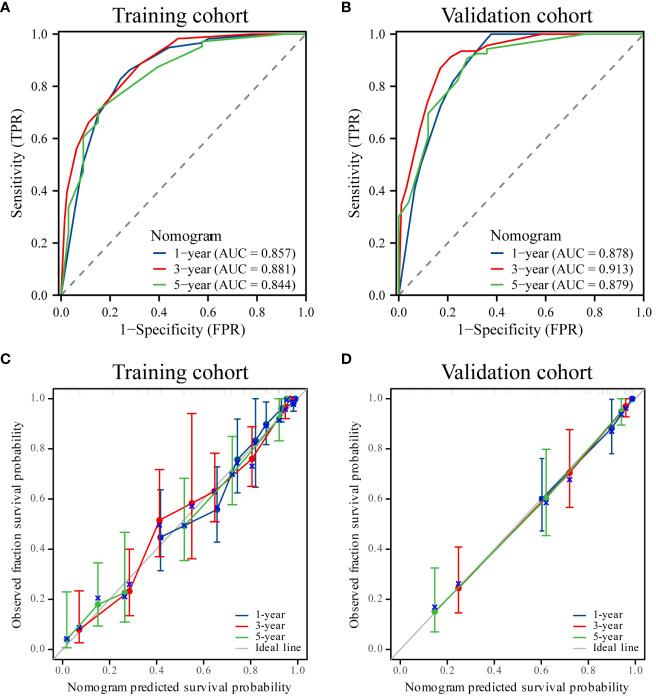
ROC curves and calibration curves. **(A, B)** ROC curves of the nomogram for 1-, 3- and 5-year overall survival (OS) in the training and validation cohorts. **(C, D)** Calibration curves showing the probability of 1-, 3-, and 5-year OS between the nomogram prediction and actual observation. The prediction probability of the nomogram for OS was plotted on the X-axis, and the actual probability was plotted on the Y-axis.

Utilizing the identified independent prognostic factors(sex, AJCC TNM Stage, FIDA level at el.), we developed a nomogram to predict patient OS at 1, 3, and 5 years. Nomograms are an excellent visualization tool to quantify the results of Cox regression equations. The nomogram indicated that a higher total score corresponded to a lower survival rate, as represented in **Figure 5**. Time-dependent ROC curve analysis showed that the AUC of the nomogram in the training cohort at 1, 3, and 5 years were 0.857, 0.881, and 0.844, respectively (**Figure 6A**), and the AUC values of the validation cohort were 0.878, 0.913, and 0.879 (**Figure 6B**). These results demonstrate the highly accurate prediction of patient outcomes by the model. We further performed discrimination and calibration of the nomogram. The C-index was 0.821 in the training cohort and 0.857 in the validation cohort, respectively (**Table 4**). The calibration curves for 1, 3, and 5-year OS in both the training and validation cohorts displayed a remarkable congruence between the predicted OS and the actual observed OS (**Figures 6C, D**).These results indicate that the model exhibits relatively accurate discrimination ability.

**Table 4:** C-index of the nomogram models.

Cohort	C-index	95% CI
Training	0.821	0.805–0.836
validation	0.857	0.837–0.877

**Figure 7:**
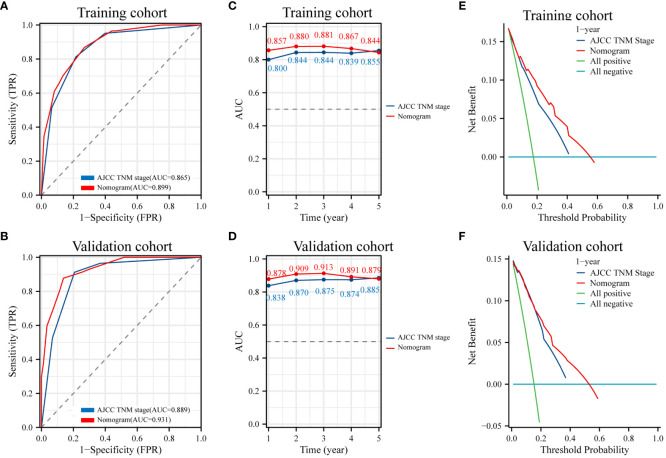
ROC curves and DCA analyses of the nomogram model and AJCC TNM stage. **(A, B)** Diagnostic ROC curves of the nomogram and AJCC TNM stage in the training and validation cohorts. **(C, D)** Time-dependent AUC curves for the training and validation cohorts of the nomogram and AJCC TNM stage. **(E, F)** Decision curve analysis (DCA) to assess the clinical decision benefit of the training and validation cohorts of the nomogram and AJCC TNM stage. AJCC, the American Joint Committee on Cancer. TNM, tumor, node, metastasis.

To evaluate the prognostic value of the nomogram and AJCC TNM stage for patients, ROC curves were used for further analysis. The AUC in the nomogram for OS is 0.899 in training cohort, and the AJCC TNM stage is 0.865 (**Figure 7A**). Similarly, the AUC in the nomogram for OS is 0.931 in validation cohort, and the AUC in the AJCC TNM stage for OS is 0.889 in validation cohort (**Figure 7B**). Time-dependent ROC curve analysis showed that the AUC of the nomogram for survival prediction at 1, 3, and 5 years in the training cohort and the validation cohort were higher than those of the AJCC TNM stage (**Figures 7C, D**). It was considered that the predictive efficacy of the nomogram was better than that of the AJCC TNM stage. Additionally, DCA showed that the nomogram had better clinical utilization than the AJCC TNM staging system at 1 year (**Figures 7E, F**). These results confirmed that the FIDA-based nomogram can accurately and effectively predict survival in NSCLC.

## Discussion

Lung cancer, recognized as the predominant cause of cancer-related mortality, continues to pose a significant global health challenge. Despite advancements in treatment approaches, there has been limited success in extending survival times. Consequently, the exploration of biomarkers related to prognosis is pivotal in devising tailored treatment strategies, ultimately enhancing clinical outcomes for lung cancer patients. In this research, we developed a novel prognostic index named FIDA, displaying promise for risk stratification and prognosis prediction in NSCLC cases. Leveraging Lasso regression, we identified eight prognostic indicators related to OS - leukocytes, neutrophils, monocytes, PLR, albumin, prealbumin, fibrinogen, and D-dimer to establish a comprehensive 8-index risk model. This model was recognized, for the first time, as an independent prognostic factor for OS in NSCLC patients. To the best of our knowledge, this is the inaugural study highlighting the prognostic importance of FIDA in individuals with NSCLC.

Systemic inflammation, a key interaction between the host and tumor in patients with cancer, plays a critical role in the development, progression, metastasis, and treatment resistance of cancer (20). Peripheral blood tests conducted at diagnosis or prior to treatment can serve as indicators of the tumor’s inflammatory state. In many cancers, including NSCLC, peripheral blood cells are crucial prognostic elements. Specifically, neutrophils in NSCLC secrete IL-6 and IL-12, fostering the tumor’s inflammatory microenvironment. This, in turn, stimulates neutrophil proliferation, perpetuating a harmful cycle (21). Monocytes, macrophage precursors, also support tumor development and its inflammatory milieu, thereby facilitating tumor invasion (22). The prognostic significance of the PLR, a systemic inflammation marker derived from platelet and lymphocyte counts, has been explored in various cancers such as cholangiocarcinoma (23) and gastric cancer (24). Oncology patients frequently experience malnutrition, which not only complicates clinical decision-making in cancer treatment but also heightens complication and mortality rates, diminishes patient quality of life, and impacts clinical outcomes (25). Albumin serves as a valuable nutritional marker that also neutralizes pro-inflammatory stimulants in the body. Its role in evaluating the prognosis of NSCLC patients is significant. A clinical investigation by Kazuki et al. identified Alb as an independent prognostic factor in NSCLC patients undergoing PD-1 inhibitor therapy (26). Prealbumin, a smaller protein, has gained recognition as both a nutritional and prognostic marker (27). Fibrinogen primarily facilitates the formation of metastases from circulating tumor cells (28, 29). Moreover, activation of the hemostatic system, particularly its heightened activation, is linked to advanced tumor stages, adverse outcomes, and poor prognosis in various solid tumors (30, 31). Elevated plasma D-dimer levels before treatment are an unfavorable prognostic marker for several malignancies (32, 33).

In this research, a preliminary LASSO Cox regression analysis was conducted on 20 inflammatory trophic factors to identify eight key prognostic indexes related to OS in NSCLC patients, including leukocytes, neutrophils, monocytes, PLR, albumin, prealbumin, fibrinogen, and D-dimer. Following this, the FIDA prognostic index risk model was established. FIDA demonstrated notable diagnostic value for patients, as evidenced by prognostic ROC curves, which indicated its strong predictive capability for patient outcomes at 1, 3, and 5 years (validation cohort AUC values: 0.789, 0.817, and 0.821, respectively). Kaplan-Meier survival analyses revealed that patients with high FIDA scores had significantly lower OS compared to those with low FIDA in both the training and validation cohorts (P<0.001). Further analysis of FIDA’s prognostic utility across different subgroups showed its relevance in terms of gender, age, and smoking history. Multivariate regression analysis pinpointed gender, AJCC TNM stage, and FIDA as independent prognostic factors, with FIDA emerging as a risk factor for NSCLC patients. Moreover, a column-line graph constructed based on these independent factors exhibited a C-index of 0.857 and impressive diagnostic efficiency, with AUC values for 1-, 3-, and 5-year diagnostic ROC curves in the validation cohort being 0.878, 0.913, and 0.879, respectively. Calibration curves for 1-, 3-, and 5-year OS displayed a high degree of concordance between predicted and actual observed OS in both cohorts. Notably, when comparing the efficacy of the prognostic nomogram to AJCC TNM staging system using time-dependent AUC curves, nomogram’s accuracy was found to be superior in both the training and validation cohorts. The study underscores that even minor variations in biomarkers can significantly influence the diagnostic accuracy of predictive markers. The FIDA-based nomogram is a more comprehensive and meaningful predictor.

Nevertheless, the study is subject to certain limitations: primarily, its nature as a single-center retrospective study raises concerns regarding a potentially limited study population, selection bias, insufficient follow-up duration, and genetic variability among participants. Additionally, the absence of a standardized threshold for FIDA may contribute to discrepancies in outcome assessments, as various prior studies have employed differing statistical approaches to determine this threshold. Consequently, there is a need for future research involving prospective, large-scale, multicenter randomized controlled studies to further substantiate the clinical predictive value of FIDA in NSCLC patients.

## Conclusions

In this study, we found for the first time that FIDA is a novel, simple, and effective prognostic marker for NSCLC. Fida-based nomogram can accurately and effectively predict individual survival of NSCLC. The prediction model shows good discrimination and calibration through internal and external validation, thus improving the clinical decision making ability of clinicians.

## Data availability statement

The raw data supporting the conclusions of this article will be made available by the authors, without undue reservation.

## Ethics statement

The studies involving humans were approved by the Ethics Committee of Qingdao Municipal Hospital. The studies were conducted in accordance with the local legislation and institutional requirements. The ethics committee/institutional review board waived the requirement of written informed consent for participation from the participants or the participants’ legal guardians/next of kin because the requirement for informed consent was waived for the retrospective study.

## Author contributions

HQ: Conceptualization, Data curation, Formal analysis, Investigation, Methodology, Project administration, Software, Supervision, Validation, Visualization, Writing – original draft, Writing – review & editing. YF: Conceptualization, Data curation, Formal analysis, Investigation, Methodology, Project administration, Software, Supervision, Validation, Visualization, Writing – original draft, Writing – review & editing. XH: Conceptualization, Data curation, Formal analysis, Investigation, Methodology, Software, Validation, Writing – original draft. HT: Data curation, Funding acquisition, Investigation, Project administration, Resources, Supervision, Validation, Visualization, Writing – review & editing.
